# The distribution of *Dermacentorreticulatus* in the Czech Republic re-assessed: citizen science approach to understanding the current distribution of the *Babesiacanis* vector

**DOI:** 10.1186/s13071-022-05242-6

**Published:** 2022-04-18

**Authors:** Ondřej Daněk, Kristýna Hrazdilová, Dominika Kozderková, Daria Jirků, David Modrý

**Affiliations:** 1 Department of Pathology and Parasitology, Faculty of Veterinary Medicine, University of Veterinary Sciences, Brno, Czech Republic; 2 CEITEC VETUNI, University of Veterinary Sciences, Brno, Czech Republic; 3 Faculty of Medicine in Pilsen, Biomedical Center, Charles University, Plzeň, Czech Republic; 4 Faculty of Science, University of South Bohemia, České Budějovice, Czech Republic; 5 Institute of Parasitology, Czech Academy of Sciences, České Budějovice, Czech Republic; 6 Department of Botany and Zoology, Faculty of Science, Masaryk University, Brno, Czech Republic; 7 Department of Veterinary Sciences, Faculty of Agrobiology, Food and Natural Resources (CINeZ), Czech University of Life Sciences, Prague, Czech Republic

**Keywords:** *Dermacentorreticulatus*, *Babesiacanis*, Citizen science, Czech Republic, Geographic distribution, Europe

## Abstract

**Background:**

The range of the ornate dog tick *Dermacentor* *reticulatus* is rapidly expanding in Europe. This tick species is the vector of canine babesiosis, caused by *Babesia* *canis*, and also plays a role in the transmission of *Theileria* *equi* and *Babesia* *caballi* in equids.

**Methods:**

The geographic range of *D.* *reticulatus* in the Czech Republic was re-assessed, and an up-to-date distribution map is presented based on material and data obtained during a nationwide citizen science campaign. Received and flagged individuals of *D.* *reticulatus* were also analysed for the presence of *B.* *canis* DNA.

**Results:**

In striking contrast to historical records, *D.* *reticulatus* was found in all regions of the Czech Republic, with most reports coming from the southeast and northwest of the country. Between February 2018 and June 2021, the project team received 558 photo reports of ticks and 250 packages containing ticks. Of the former, 71.1% were identified as *Dermacentor* sp. with the remainder identified as *Ixodes* sp., *Haemaphysalis* sp., *Argas* sp. or *Hyalomma* sp. The majority of specimens in the subset of ticks that were received (*N* = 610) were *D.* *reticulatus* (*N* = 568, 93.7%), followed by *Ixodes* *ricinus* and *Hyalomma* spp. A total of 783 adult *D.* *reticulatus*, either received (568) or collected by flagging (215), were tested for the presence of *B.* *canis* DNA using species-specific nested PCR targeting part of the* 18S* rRNA gene; *B.* *canis* DNA was demonstrated in 22 samples (2.81%).

**Conclusions:**

The continuous spread of *D.* *reticulatus* in the Czech Republic was documented in this study. In addition, DNA of *B.* *canis* was also detected in a number of ticks, suggesting the establishment of *B.* *canis* in the Czech Republic. These results suggest that veterinarians need to consider the possibility of canine babesiosis even in dogs without a history of travel.

**Graphical Abstract:**

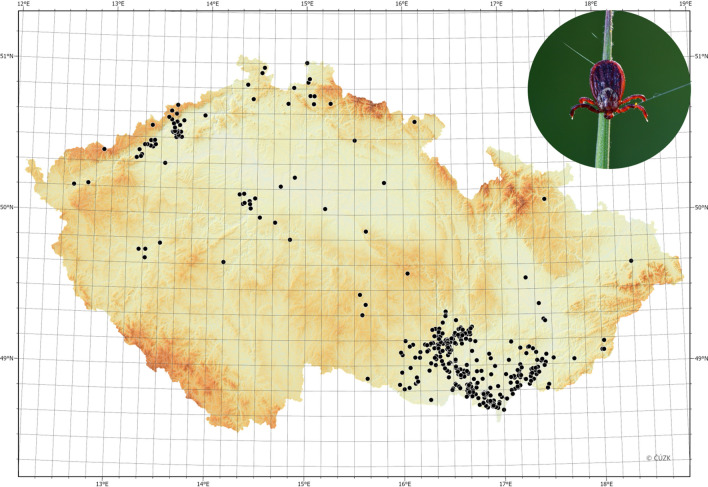

## Background

Environmental and societal changes attributable to the climate change have a significant impact on the spread of vector-borne diseases and their arthropod vectors [1]. There are also species in the tick fauna whose ranges are changing rapidly. *Rhipicephalus* *microplus* is a prominent example of a tick associated with cattle that has invaded both tropical and subtropical areas [2, 3]. In general, changes in the geographic ranges of ticks occur in two ways, although these may overlap. The first is long-distance dispersal followed by local dispersal. Range expansion in this way is usually associated with transboundary animal movements. An example of such range expansion is the Asian tick *Haemaphysalis* *longicornis* and its recent spread into the northeastern and southeastern regions of the USA [4, 5]. The second form of range expansion is a gradual expansion of geographic range (usually to higher altitudes or latitudes) due to environmental and climate changes associated with host migration. The ticks *Amblyomma* *americanum* and *A.* *maculatum* have shown this type of expansion in North America [6, 7].

The most prominent example of a tick showing a rapidly expanding distribution in Europe is the ornate dog tick *Dermacentor* *reticulatus* [8], one of two members of the genus *Dermacentor* distributed in Europe [9]. Its geographic range is highly focal and discontinuous, and consists of two main macroregions [10, 11]. The first is the western European macroregion, which extends from northern Spain to western Poland, France, with isolated foci in the Netherlands, Belgium, Hungary, the Czech Republic, Slovakia and Germany. The second is the eastern European macroregion, which extends from eastern Poland to the Baltic States and Russia [9, 12]. The ornate dog tick occurs much further north than its congener *Dermacentor* *marginatus*, reaching the British Isles [13], northern Germany [14], Poland [15] and the Baltic States [16–18], and it may be also spreading southward [19, 20].

*Dermacentor* *reticulatus* is a three-host tick that circulates among rodents (larvae and nymphs) and larger carnivores and herbivores (adults). Consequently, it is associated with the transmission of a number of tick-borne pathogens throughout its range, including tick-borne encephalitis virus *Rickettsia* *raoultii* and *Rickettsia* *slovaca* [8, 21]. *Dermacentor* *reticulatus* is the only vector of canine babesiosis, which is caused by *Babesia* *canis*. In temperate Europe this tick may also play a role in the transmission cycle of *Theileria* *equi* and *Babesia* *caballi*, the two piroplasmids found in equids [8, 22].

**Fig. 1:**
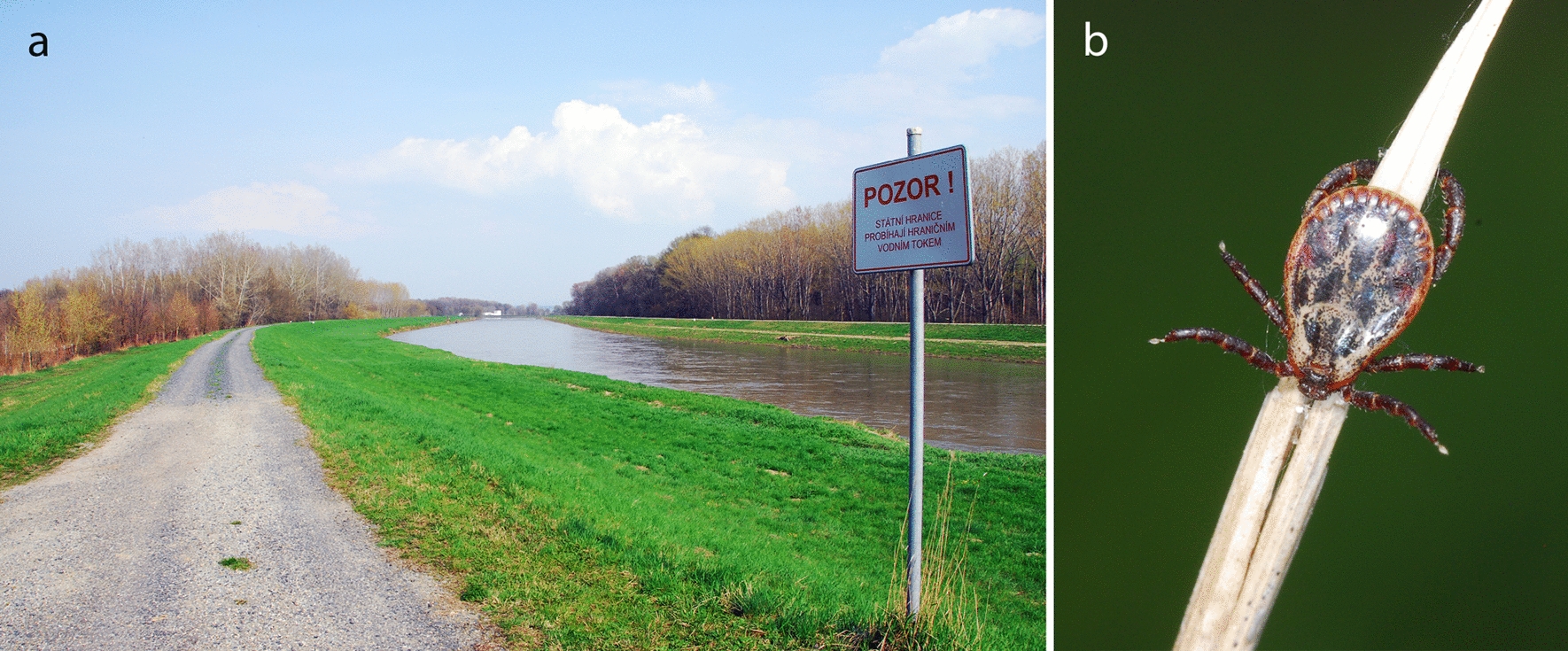
Typical habitat of *Dermacentor* *reticulatus* along the Morava River at the border with Slovakia (**a**) and close-up picture of questing male in the same locality (**b**)

**Fig. 2:**
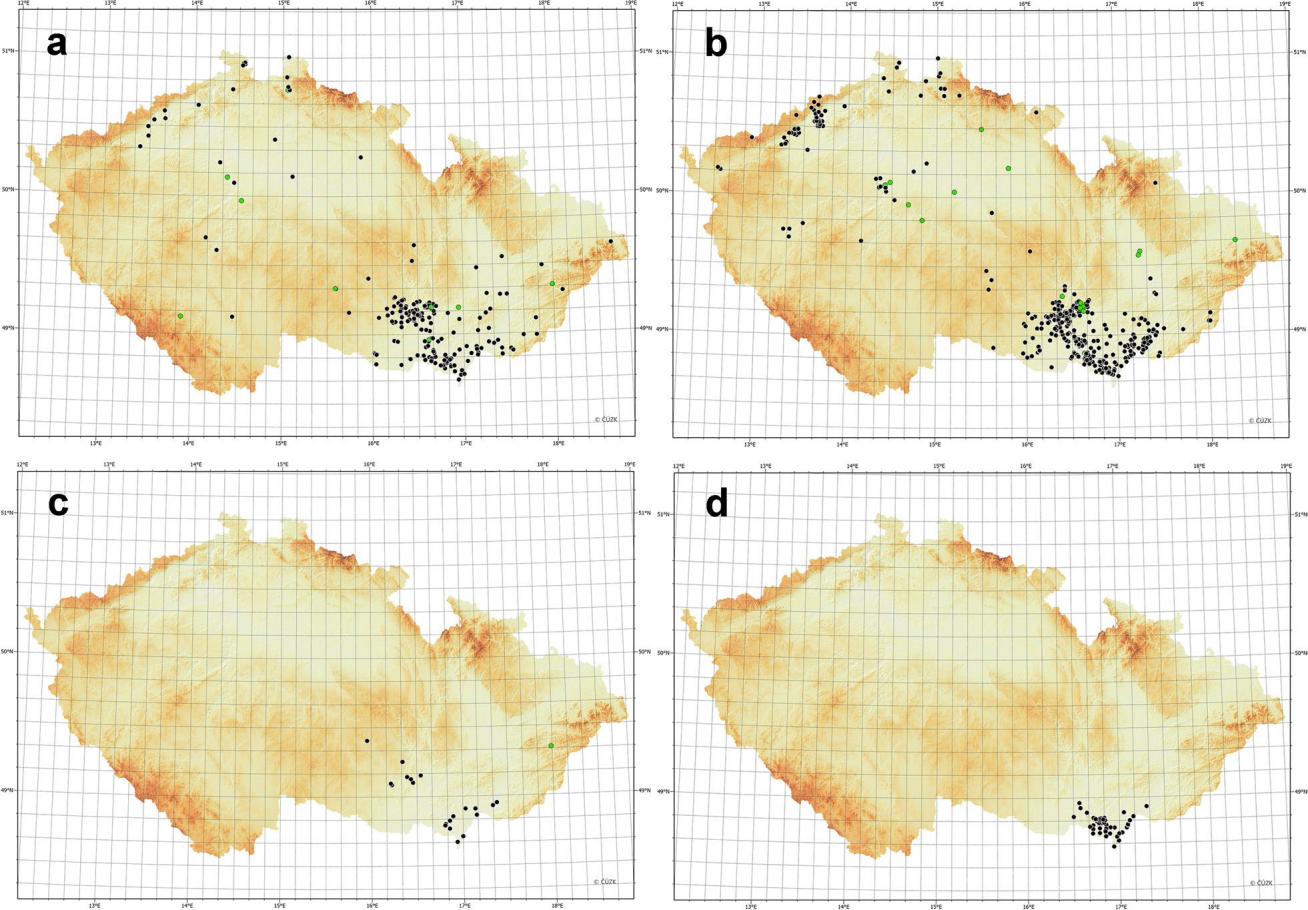
Georeferenced findings of *D.* *reticulatus* and *Babesia* *canis* in the Czech Republic. **a** Morphologically confirmed records of *D.* *reticulatus* based on the received ticks, **b** findings based on photo reports (tentatively identified as *D.* *reticulatus*), **c** finding of *B.* *canis* DNA as revealed by PCR targeting partial* 18S* rDNA, **d** previous reports of *D.* *reticulatus* extracted from Široký et al. [26]. Green colour in **a**–**c** indicates reported travel history (of dogs) in the 2 weeks prior to the observation

The occurrence of *D.* *reticulatus* in the Czech Republic was described as early as 1952 by Rosický [23] in the southeastern corner of the country in Tvrdonice (Břeclav district), and it presence was confirmed in the 1970s in the same region [24, 25] (Fig. 1). In a recent comprehensive study carried out in 2009–2010 [26], the tick was found in 46 out of 100 surveyed sites in the South Moravia region, more or less defining the distribution limits of *D.* *reticulatus* in the Czech Republic (Fig. 2d). Until recently, babesiosis caused by *B.* *canis* was considered to be an imported disease in the Czech Republic. The presence of *B.* *canis* DNA was detected for the first time in the Czech Republic in 2017 in shelter dogs in the core area of *D.* *reticulatus* distribution in the South Moravia region, and the first clinical autochthonous case of *B.* *canis* was diagnosed 1 year later in a non-travelling dog in the South Moravia region [27, 28].

Lewis et al. [29] reviewed the role of citizen science in tick research and presented an analysis of the advantages and disadvantages of this strategy. Recent nationwide mapping of ticks in the Netherlands [30] and Germany [14] are examples of different citizen science approaches that have been used in European projects. While the Dutch study used trained volunteers to collect ticks in defined areas, the latter study collected *Dermacentor* spp. ticks sent in by mail by citizens.

The aim of our study was to redefine the geographic range of *D.* *reticulatus* in the Czech Republic and to present an up-to-date distribution map based on georeferenced material obtained during a nationwide citizen participatory science campaign. We also present data on the prevalence of *B.* *canis* DNA in received and flagged ticks.

## Methods

### Citizen science

The citizen science project “Najdi pijáka” (literally translated from Czech as: “Find the ornate tick”) was launched by the project team in 2018. An informative website (www.najdipijaka.cz) for the public was created in the Czech language and launched in late February 2018 with a contact email address and subsequent Facebook account (FB) for receiving photo reports and communicating with individuals who provide tick records. The project website included three parts: (i) a brief description of the project aims and research team; (ii) the basic biology of *D.* *reticulatus* and key morphological features that allow easy differentiation between ticks of the genera *Ixodes* and *Dermacentor* in Central Europe; and (iii) information on how to report observations of target ticks and how to safely turn in collected specimens. The final section included simple instructions on how to take photographs, the accompanying data that were necessary (date of find/collection, location, host), tips on how to send the ticks and records by mail, email or via a social media account and contact information. A key component of the website was a map showing current distribution data. To maximise public awareness, the project was announced through various media sources during its duration (February 2018–June 2021). The releases always included images comparing *D.* *reticulatus* with *Ixodes* *ricinus* and a link to the project website. The importance of *D.* *reticulatus* as a vector of canine babesiosis was emphasised in the press releases, which were primarily directed to dog owners. The research team informed the participants about the identification of the received ticks (both physical and photo reports) and shared the results of molecular detection of *B.* *canis* in the received ticks. Through the email account and FB, the research team communicated with participants and answered various questions related to *D.* *reticulatus* and *B.* *canis* infection. All participants either provided or were asked to provide additional information for the report to be added to the data collection. These consisted of: (i) site of discovery (either GPS coordinates or address); (ii) date of discovery; (iii) host association; and (iv) travel history of the associated/perceived host in the past 14 days.

### Tick collection and identification

To expand the dataset of ticks used for *B.* *canis* detection, material collected within the framework of the citizen science project was supplemented with ticks collected by the project team using a white cotton flag (79 × 94 cm) attached to a 150-cm wooden pole [31]. The collection sites were based on data obtained from the citizen science part of the project and from published reports on the distribution of *D.* *reticulatus* [26]. A total of seven collections were made at five sites in the southeastern part of the Czech Republic (South Moravia region) in April (5 collections) and September (2 collections) 2019. Only adult ticks were collected.

Tick reports consisting of photographs (photo reports) were identified to the genus level based on general appearance, body and leg shape, and pattern of scutum. All ticks received and collected were identified to the species level based on their morphology. The identification of the *Dermacentor* ticks received/collected was performed using the key to the species of genus *Dermacentor* in Europe and Northern Africa [18] by microscopic observation (model SZ51; Olympus Corp., Tokyo, Japan). A tick was placed dorsal side up on a microscopic slide with forceps, and the species was determined by the presence of a distinct, posteriorly directed spur on the dorsal palp article II [18].

### DNA isolation and *B. canis* detection

Ticks were stored in individual tubes containing DNase-free water in the freezer before DNA isolation. Each tick was cut in half lengthwise. DNA was extracted from one half using the Exgene Cell SV Mini 250p Kit (GeneAll, Seoul, Korea) according to the standard protocol for animal tissues, with 50 µl of elution buffer added in the final step. The unused tick half was stored under the above conditions for future experiments or re-analysis.

For detection of *B.* *canis* in isolated DNA, part of the small subunit of the* 18S* ribosomal RNA (rRNA) gene was targeted using nested PCR. The 376-bp fragment of* 18S* rDNA was amplified using primers Bc_F1, GR2 and Bc_F2, Bc_R1 [32, 33]. The first round of PCR was prepared in a total volume of 15 µl, which included 7.5 µl of 2× PCRBIO Taq Mix Red (PCR Biosystems, London, UK), 7.5 pmol of each primer (Bc_F1 and GR2) and 2 µl of template DNA. PCR conditions for the first round consisted of an initial denaturation at 95 °C for 1 min, followed by 35 cycles of denaturation at 95 °C for 15 s, annealing at 50 °C for 15 s and extension at 72 °C for 5 s, with a final extension at 72 °C for 5 min. The second round of PCR was performed in a total volume of 25 µl, which included 12.5 µl of 2× PCRBIO Taq Mix Red, 10 pmol of each primer (Bc_F2 and Bc_R1) and 1 µl of the PCR product from the first round). The PCR conditions for the second round consisted of an initial denaturation at 95 °C for 1 min, followed by 35 cycles of denaturation at 95 °C for 15 s, annealing at 53 °C for 15 s and extension at 72 °C for 15 s, with a final extension at 72 °C for 5 min. The PCR products were visualised in a 2% agarose gel stained with Midori Green Advance DNA Stain (Nippon Genetics Europe, Düren, Germany). All samples that yielded an amplicon of the appropriate size were excised and purified using the Gel PCR DNA Fragments Extraction Kit (Geneaid, New Taipei City, Taiwan). The purified PCR products were sent for commercial Sanger sequencing (Macrogen Europe, Amsterdam, The Netherlands). Geneious Prime software (Biomatters, Auckland, New Zealand) was used to assemble and analyse the sequences obtained.

## Results

### Identification of ticks received

In total, the project team received 558 photo reports and 250 packages containing ticks (Table 1); 71.1% of the photo reports were identified as *Dermacentor* sp. and the remaining ticks on the photo reports were identified as *Ixodes* sp., *Hyalomma* sp., *Haemaphysalis* sp. and *Argas* sp. In the subgroup of identified received ticks (*N* = 610), the majority of ticks were identified as *D.* *reticulatus* (*N* = 568, 93.1%), followed by *Ixodes* *ricinus* and *Hyalomma* spp. (Table 1). The number of received ticks ranged from 1 to 102 ticks per package.

**Table 1:** Records of ticks received as a result of the citizen science campaign either as photographs (photo reports) or as ticks delivered by post (received ticks)

Type of report	*Dermacentor*	*Ixodes*	*Hyalomma*	*Haemaphysalis*	*Argas*
Photo report^a^	397	140	3	9	9
Ticks received^b^	568 (212)	30 (26)	12 (12)	0	0

^a^Numbers indicate the record numbers; it was not possible to evaluate the real number of observed ticks

^b^Numbers in brackets indicate the number of reports

### Geographic distribution of *D. reticulatus*

The site of discovery was indicated in all 609 reports of *Dermacentor* ticks (see Fig. 2a–c, with the location of tick discovery indicated in green together with reported travel history (of dogs). As the determination of the ticks to species level was possible only in physically received specimens (568 ticks in 212 records), distribution data for physically received ticks and photo records are shown separately in the maps shown in Fig. 2 and 2b, respectively. *Dermacentor* ticks were reported in all 14 regions of the Czech Republic. The majority of findings (*N* = 429, 70.4% of reports) were from the South Moravia region; this part of the distribution is termed the core area hereafter. The second most represented region was the Ústí nad Labem region in Northern Bohemia (*N* = 57, 9.4% of reports). However, individuals of *D.* *reticulatus* were reported from the whole Czech Republic (Fig. 2a, b).

### Seasonality of *D. reticulatus* findings and host association

**Fig. 3:**
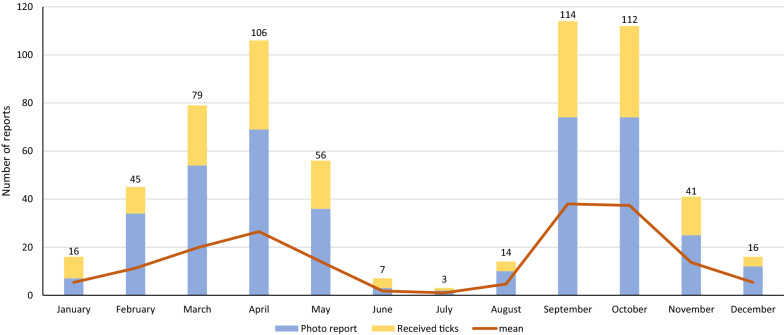
Seasonal activity of *D.* *reticulatus*. Cumulative number of *Dermacentor* findings reported through the citizen science project in each month (February 2018–June 2021) shows bimodal seasonal distribution and occurrence in winter months. As not all the months were equally represented “Mean” refers to the number of reports in that month divided by the number of that specific month

The date of collection/observation was available for all 609 *Dermacentor* observations reported through this citizen science project. Analysis of the monthly tick occurrence data (Fig. 3) reveals an obvious seasonality, with peaks in the spring and early fall. Host association information for the reported *Dermacentor* ticks was available for 463 of the 609 records (Table 2). Most of these finds (80.6%) were reported from dogs, followed by finds from humans. A minority of reported *D.* *reticuatus* ticks were observed on other animals or in the environment. The number of ticks observed per host ranged from 1 to 40 individuals, with several ticks occurring almost exclusively on dogs. Of the 373 reports of ticks on dogs, 29 indicated recent travel of the dog within the Czech Republic (24), Slovakia (4) and Germany (1).

**Table 2:** Reported host association of *Dermacentor* findings

Hosts	Findings
Dog	373
Human	68
Horse	5
Cat	2
Lama	1
Environment	18
Data not provided	149

Numbers indicate the number of photo record/parkages; for photo records, the real number of ticks could not be evaluated

### Occurrence of *B. canis* in examined ticks

All physically received (568) or flagged (215) *D.* *reticulatus* individuals were tested for the presence of *B.* *canis* DNA. The amplicon of the expected size (376 bp) was detected in 22 samples (2.81%). Nineteen positive ticks were sent in by citizen scientists, and three positive ticks (2 females, 1 male) were collected by flagging. Of the 19 ticks sent in, 17 were found on dogs (11 males, 6 females) and two were found on a person (both females). Based on only ticks from the core area, there were 18 positive results out of 566 tests (3.18%). Sequencing resulted in high-quality, 100% identical sequences of 320–357 bp (GenBank acc. no. OK135945, this study). The BLAST analysis confirmed 100% identity and query cover of the sequence to more than 50 different *B.* *canis* sequences from different geographic regions and/or hosts, including sequences from the Czech Republic and surrounding countries (e.g. MK024714 from a domestic dog, Poland; KY693669 from a red fox, Austria; KY021188 from a domestic dog, Czech Republic).

## Discussion

Citizen science is a research concept based on the concept of using interested citizens to investigate various scientific topics. Field biology and wildlife monitoring are one of the areas where citizen science has gained prominence in recent decades [34]. *Dermacentor* spp. are highly recognisable ticks, so we were able to use both photographic reports and physically received ticks in our geographic survey. The ability to participate in this study by submitting only a photograph facilitated the participation of a large number of interested individuals and increased the reliability of the data collected. Although tick identification can be difficult for untrained volunteers, participants in our study were able to identify ticks in most cases and contributed immensely to mapping the distribution of *D.* *reticulatus* (Table 1).

Since only the sent in and flagged ticks could be determined to species level, all photo reports were considered to be *Dermacentor* sp. While the genus *Dermacentor* is represented in Europe by *D.* *reticulatus* and *D.* *marginatus* [9], there are currently no records of *D.* *marginatus* in the Czech Republic. The nearest known populations to the Czech Republic are those in Germany, in the Rhine and Main valleys [35], and in Slovakia [25, 36]. Since no significant spread of *D.* *marginatus* has been observed in the German population [14] and there are no recent data indicating dispersal in Slovakia, it is very likely that all photo reports of *Dermacentor* from the Czech Republic are *D.* *reticulatus*.

The most recent data published by Široký et al. in 2011 [26] demonstrated the occurrence of *D.* *reticulatus* only in the South Moravia region. The majority of the records collected in our study are also from this region. However, according to our data, the population of *D.* *reticulatus* has expanded to an area between 16.2–17.5 °E and 48.7–49.25 °N, making it the core area of distribution in the Czech Republic (the core area). Participants in our citizen science campaign recorded *D.* *reticulatus* ticks in all regions of the Czech Republic, with a clustering of reports in the northwestern part of the country, indicating that a subpopulation has been established in this region. Most other reports outside the core area were presented as sporadic findings. A recent travel history of dogs was reported for 29 of these findings, and 15 dog owners reported travelling to the core area of *D.* *reticulatus* in the Czech Republic prior to tick observation (Fig. 2a–c). It would appear that the movement of dogs is a route by which *D.* *reticulatus* spreads in the Czech Republic. According to our observations, most *Dermacentor* ticks observed on dogs were found as freely moving adult individuals. As such, these ticks are not affected by oral ectoparasiticides based on isoxazolines and avermectins and can spontaneously leave the host to infest new areas. In the same way, detached, engorged females can establish new populations. Therefore, a more thorough survey of areas where the presence of *D.* *reticulatus* has been repeatedly reported is needed to assess the distribution of this tick species in the Czech Republic and the resulting risk of canine babesiosis transmission.

Currently, *D.* *reticulatus* is present in all neighbouring countries of the Czech Republic [9]. In Slovakia it is a common tick [37, 38], and from Austria there are records of observations in the northeastern part of the country and around Vienna [39, 40]. The core population of *D.* *reticulatus* in the Czech Republic is likely associated with the distribution area in these two countries. Similarly, the distribution of *D.* *reticulatus* in Poland [15, 41] and Germany [14, 42] is well documented. In particular, the German foci in Saxony are located in close proximity to the Czech border. It is therefore quite possible that some (if not all) observations of the ornate dog tick in the northwestern part of the Czech Republic are due to dispersal from Germany (and possibly Poland) rather than to dispersal of the core population in the southeastern part of the Czech Republic.

The spread of *D.* *reticulatus* is commonly attributed to climatic and socioeconomic changes as suitable habitats for the ticks or their hosts emerge due to a warmer climate, changes in agricultural use of the landscape and/or increased migration and colonisation of vacant territories by vertebrate hosts [42, 43]. The ecology of ticks and their preferred hosts may play a critical role in range expansion. *Dermacentor* *reticulatus* is a three-host tick with a shorter life-cycle and higher cold tolerance compared to *I.* *ricinus* [18, 44]. Larvae and nymphs usually feed on smaller mammals and occasionally birds, while adult ticks use larger herbivores and carnivores as hosts [45, 46]. Our data show that dogs are the most important host species of the ornate dog tick (Table 2); however, these findings are heavily impacted by the citizen science campaign. Dogs play an important role in the life-cycle of *D.* *reticulatus*, especially in urban areas [8], but other large mammals, such as roe deer *Capreolus* *capreolus*, red deer *Cervus* *elaphus* and wild boar *Sus* *scrofa* are also involved in the life-cycle of this tick [47]. The grey wolf *Canis* *lupus*, Eurasian golden jackal *Canis* *aureus* and the elk *Alces* *alces* can all harbour *D.* *reticulatus*, and all three of these species also have great migratory potential [48–51]. In the last decade, the wolf population has become established in the northern parts of the Czech Republic [52]. Genetic analyses show that these wolves belong to the so-called lowland population that is spreading into the Czech Republic from Germany and Poland [53]. During necropsies of wolf carcasses found in the northern part of the Czech Republic (data not shown; University of Veterinary Sciences Brno), we found high numbers of *D.* *reticulatus* in three cases, suggesting that these predators may play a role in the spread of this tick. The spread of the *D.* *reticulatus* population in the Czech Republic is likely due to a combination of several factors: (i) direct links to nearby populations in Germany and Poland [14, 41]; (ii) migration of host species, especially large carnivores; (iii) movement of resident dogs with their owners across the country.

*Dermacentor* *reticulatus* is the only confirmed definitive host of *B.* *canis* [22]. Canine babesiosis caused by *B.* *canis* is becoming more common in all neighbouring countries of the Czech Republic [54–56]; however, cases in the Czech Republic are still very rare. The only documented autochthonous clinical case of canine babesiosis was recently reported from the core area of *D.* *reticulatus* in the Czech Republic [28]. All ticks positive for *B.* *canis* DNA were reported from the southeastern part of the Czech Republic, mainly from the core population; however, this may be due to the relatively small number of samples from other parts of the country. Although 17 of 22 positive *D.* *reticulatus* were found on dogs in our study, none of the owners reported clinical signs attributable to canine babesiosis. One explanation is that none of the positive female ticks were engorged. In addition, most of these ticks were found crawling on the host shortly after a walk, indicating that there was insufficient time for transmission. Since none of these dogs were tested for the presence of *B.* *canis*, they could be asymptomatic carriers of the disease [57]. *Babesia* *canis* is also known to be a parasite in the Eurasian golden jackal [27] and grey wolf [58], and a recent study from Poland has also demonstrated the presence of *B.* *canis* DNA in the red fox *Vulpes* *vulpes* [59]. Because all of these canids co-occur in the Czech Republic, it is possible that they play a role in maintaining *B.* *canis* foci, as the number of infected dogs is minimal. Transovarial transmission of *B.* *canis* must also be considered. *Dermacentor* *reticulatus* could also be a vector of *B.* *caballi* and *T.* *equi* [8, 60, 61], both of which were recently detected in the Czech Republic [62]. Although the autochthonous nature of equine piroplasmosis remains to be confirmed, the growing population of *D.* *reticulatus* may play a role in creation of an environment suitable for the persistence of endemic transmission cycles of these piroplasmids.

*Dermacentor* *reticulatus* is known for its seasonal activity [63]. The data we obtained show year-round activity with peaks in the spring and early fall. Interestingly, we received more reports from the winter months (December to February) than from the summer months (June to August), showing a similar pattern as described by other authors [64]. These results have striking practical implications for protecting dogs from this tick as they suggest that preventive measures in the form of repellents or acaricides should be applied at least from early spring to late fall and ideally, particularly in lowland regions, throughout the year. Although we detected the presence of *B.* *canis* only in ticks from the core population, veterinarians should be aware of the presence of *B.* *canis* in the Czech Republic and consider this disease regardless of the travel history of their canine patient, as a continuous spread of *B.* *canis* and its vector in the Czech Republic is expected.

## Conclusions

The continuous spread of *D.* *reticulatus* in the Czech Republic was documented in this study by a citizen science project. The core population of this tick, described in a previous study [26], has significantly expanded its range since the publication of the study. In addition, *D.* *reticulatus* has been observed in all regions of the Czech Republic, with most reports coming from the southeast and northwest of the country. *Babesia* *canis* DNA was also detected in a number of ticks (2.81%), mainly in the core population of *D.* *reticulatus*, the only known vector of this piroplasm. These findings indicate that *B.* *canis* has become established in the Czech Republic, necessitating extensive research on potential reservoir hosts in the region. They also indicate that the number of endemic cases of canine babesiosis may be increasing and that veterinarians should not consider this disease to be only as an imported one. Also, based on seasonality data on *D.* *reticulatus* activity, dog owners should be advised to protect their dogs with acaricides at least from early spring to late fall but ideally throughout the year.

## Data Availability

The nucleotide sequence generated in the present study has been deposited in GenBank (https://www.ncbi.nlm.nih.gov/) under accession number OK135945. The datasets used and/or analysed during the current study are available from the corresponding author on reasonable request.
